# Immune activation and mucin dysregulation in pediatric refractory *Mycoplasma pneumoniae* pneumonia with mucus plugs

**DOI:** 10.3389/fcimb.2025.1706340

**Published:** 2026-01-05

**Authors:** Fen Liu, Qinglin Wang, Qi Cheng, Han Zhang

**Affiliations:** 1 Department of Pediatrics, Shengjing Hospital of China Medical University, Shenyang, China; 2 Department of Clinical Nutrition, Children’s Hospital Affiliated to Zhengzhou University, Henan Children’s Hospital, Zhengzhou Children’s Hospital, Zhengzhou, China

**Keywords:** BALF, epithelial injury, MUC5B, mucins, mucus plug

## Abstract

**Objective:**

Children with refractory *Mycoplasma pneumoniae* pneumonia (RMPP) experience a prolonged disease course and frequent complications, posing a serious threat to pediatric health. Bronchial mucus plugs appear to play a key role; however, their risk factors and underlying mechanisms remain incompletely defined. We sought to characterize clinical, serologic, and bronchoalveolar lavage fluid (BALF) features associated with mucus plugs in RMPP, assess serum–BALF correlations, and identify independent determinants.

**Methods:**

From January 2022 to December 2023, children meeting the criteria for RMPP and requiring bronchoscopy were enrolled, and BALF samples were collected for analysis. Patients were stratified according to the presence or absence of mucus plugs. Clinical features, serologic parameters, BALF cytokine profiles, macrolide-resistance mutations, and epithelial and mucin biomarkers were compared between groups. Correlations between systemic inflammatory markers and BALF markers were analyzed, followed by enrichment analyses of differentially expressed BALF markers. Finally, factors independently associated with mucus plug formation were identified.

**Results:**

Eighty-eight children with RMPP were enrolled, including 37 with mucus plugs and 51 without. The mucus plug group required more frequent bronchoscopy and exhibited lower lymphocyte, monocyte, platelet, total protein, albumin, sodium, and potassium levels. Conversely, C-reactive protein (CRP), procalcitonin (PCT), creatine kinase (CK), lactate dehydrogenase (LDH), and serum ferritin levels were markedly elevated. BALF analysis revealed significantly higher levels of interleukin (IL)-2, IL-4, IL-6, IL-12p70, IL-17, interferon-γ (IFN-γ), tumor necrosis factor-α (TNF-α), Krebs von den Lungen-6 (KL-6), surfactant protein A (SP-A), and MUC5B in the mucus plug group. Rates of macrolide-resistance mutations were comparable between groups. Serum LDH, CRP, and CK levels correlated positively with BALF pro-inflammatory cytokines (IL-6, IFN-γ, and TNF-α), whereas albumin and sodium levels showed inverse correlations with these cytokines and with epithelial injury markers (KL-6 and SP-A). Enrichment analyses indicated that differentially expressed cytokines and mucins were primarily involved in inflammation-related pathways and immune effector processes. In multivariable analysis, only serum total protein remained independently associated with mucus plug formation.

**Conclusions:**

Children with RMPP and mucus plugs exhibited higher BALF cytokine levels, elevated epithelial injury markers (KL-6 and SP-A), and relatively increased MUC5B expression. Lower serum total protein was independently associated with mucus plug formation. These findings refine the pathophysiological understanding of RMPP with mucus plugs and may inform targeted anti-inflammatory and mucus-modulating therapeutic strategies.

## 1 Introduction

*Mycoplasma pneumoniae* (*M. pneumoniae*) is a leading pathogen of community-acquired pneumonia (CAP) in children. Epidemics of *M. pneumoniae* pneumonia (MPP) occur worldwide every 3–7 years, accounting for more than 40% of pediatric CAP cases during outbreak years (Wang et al., 2024). While most cases of MPP are mild and self-limiting, a subset of patients develop refractory *M. pneumoniae* pneumonia (RMPP), *which is* characterized by poor response to macrolide antibiotics, *a* prolonged disease course, and frequent complications (Cheng et al., 2021; Zhang et al., 2023). The incidence of RMPP has risen in recent years, with an apparent shift toward younger age at onset, highlighting its growing impact on pediatric health (Liu et al., 2024).

At present, the management of pediatric RMPP is confronted with several challenges. Evidence supporting adjunctive corticosteroid therapy is encouraging but inconsistent, with persistent uncertainty regarding optimal timing and dosing. In addition, second-line antimicrobial agents are limited by safety concerns in pediatric populations (Gao and Sun, 2024; Li et al., 2025b). Amid this therapeutic uncertainty, airway mucus plugs have emerged as a pivotal yet underrecognized driver of persistent illness, warranting focused risk stratification and targeted intervention. Bronchial mucus plugs are a defining feature of RMPP and arise from inflammatory epithelial injury, dysregulated mucin secretion, and impaired mucociliary clearance (Xu et al., 2017; Gu et al., 2025). Although frequently underrecognized and managed inconsistently, these plugs can sustain fever and precipitate atelectasis (Xu et al., 2025). They are strongly associated with greater disease severity and are linked to delayed radiographic resolution, persistent pulmonary sequelae, and complications such as bronchial stenosis, bronchiolitis obliterans, and atelectasis (Yan et al., 2021; Xu et al., 2025; Wang et al., 2025). In severe cases, mucus plugs may cause acute airway obstruction, respiratory failure, and even life-threatening outcomes.

However, the mechanisms underlying mucus plug formation in RMPP remain incompletely defined. Macrolide resistance has been proposed as an independent risk factor for mucus plug formation, although this association has not been specifically evaluated within RMPP cohorts (Chunlian et al., 2025). Meanwhile, increasing evidence indicates that an excessive host immune response is a key driver of RMPP, contributing to persistent fever, lung injury, and impaired treatment response (Zhang et al., 2016; Li et al., 2021; Tong et al., 2022). During infection, injured airway epithelium releases biomarkers such as Krebs von den Lungen-6 (KL-6), surfactant protein A (SP-A), and short palate, lung, and nasal epithelium clone 1 (SPLUNC1), which reflect epithelial barrier disruption and impaired airway homeostasis (Keir et al., 2021; Li et al., 2025c). These epithelial alterations, together with sustained inflammatory signaling, may promote abnormal mucus secretion. However, most prior studies have focused on isolated factors rather than providing a multidimensional, cross-compartment analysis, thereby limiting mechanistic insight into mucus plug formation and its contribution to disease progression.

To address these gaps, we designed a study with four objectives: (1) to characterize the clinical profiles of children with RMPP who develop mucus plugs and compare their serologic and bronchoalveolar lavage fluid (BALF) markers with those of children without mucus plugs; (2) to evaluate cross-compartment correlations between systemic inflammatory markers and BALF markers identified as significant in univariable analyses; (3) to perform Gene Ontology (GO) and Kyoto Encyclopedia of Genes and Genomes (KEGG) enrichment analyses of differentially expressed BALF cytokine profiles, epithelial injury biomarkers, and mucins to delineate the key biological processes (BP), cellular components (CC), molecular functions (MF), and signaling pathways involved; and (4) to use multivariable regression to identify independent factors associated with mucus plug formation. By integrating these lines of evidence, we aim to refine the pathophysiological understanding of mucus plug formation in RMPP and to highlight potential therapeutic targets.

## 2 Materials and methods

### 2.1 Study design and participants

We conducted a prospective, single-center observational cohort study at Shengjing Hospital of China Medical University between January 2022 and December 2023. Consecutive patients meeting the criteria for RMPP and requiring bronchoscopy were screened. Prior to bronchoscopy, written informed consent was obtained from the guardians. During the procedure, an additional 3 mL of BALF was collected for research purposes alongside routine clinical assessments. These BALF samples were subsequently analyzed for cytokine profiles, epithelial biomarkers, mucin concentrations, and macrolide-resistance gene mutations.

The diagnostic criteria for RMPP were as follows: (1) acute respiratory manifestations such as fever, cough, or wheezing, with or without abnormal auscultatory findings (e.g., crackles or reduced breath sounds); (2) chest computed tomography (CT) revealing inflammatory infiltrates or areas of consolidation; (3) laboratory-confirmed *M. pneumoniae* infection, defined as positive immunoglobulin M (IgM) serology together with detection of *M. pneumoniae* RNA in BALF; and (4) sustained fever (axillary temperature ≥38.5°C) unresponsive to at least 7 days of standard macrolide therapy—intravenous or oral azithromycin (10 mg/kg/day for 7 days), intravenous or oral erythromycin (30 mg/kg/day for 7 days), or oral clarithromycin (10–15 mg/kg/day for 7 days)—accompanied by ongoing clinical deterioration and progressive radiological abnormalities (Expert Committee on Rational Use of Medicines for Children Pharmaceutical Group, National Health and Family Planning Commission, 2020). Conventional microbiological tests were performed concurrently to exclude co-infection with other respiratory pathogens. The exclusion criteria were as follows: (1) the presence of chronic lung disease, tuberculosis, asthma, bronchiolitis obliterans, congenital heart disease, neurological or metabolic disorders, immunodeficiency, hematologic diseases, or malignancies; or (2) incomplete clinical data. Written informed consent was obtained from all guardians.

Participants were categorized into mucus plug and non–mucus plug groups based on bronchoscopic findings. Because no universally accepted authoritative definition of bronchial mucus plugs in pediatric RMPP is currently available, the following operational criteria were adopted. Mucus plugs were defined as large, tenacious endobronchial secretions visualized during bronchoscopy that caused partial or complete obstruction of the bronchial lumen, typically forming strip- or cast-like material extending along the airway. For case classification, a minimum extent of at least one subsegmental bronchus filled or outlined by cohesive mucus was required. Cases with only scant, loose, or non-obstructive flocculent secretions were not considered to have mucus plugs and were assigned to the non–mucus plug group. Non–mucus plug findings were characterized by bronchoscopic evidence of mucosal hyperemia, edema, roughness, erosion, folds, ulcers, or only small amounts of flocculent secretions. All bronchoscopic procedures were assessed in real time by two experienced pediatric bronchoscopists, and any disagreements were resolved by review of recorded images by a third bronchoscopist until consensus was achieved.

### 2.2 Clinical data collection

Clinical data were collected on baseline characteristics (sex, age, and allergy history), disease course (peak body temperature and duration of fever and cough), clinical signs (wheezing and lung auscultation findings), radiologic findings (pulmonary consolidation and pleural effusion), procedural interventions (repeat bronchoscopy, bronchial brushing, and use of biopsy forceps), and extrapulmonary complications. Laboratory and serologic parameters included complete blood count, C-reactive protein (CRP), procalcitonin (PCT), liver and renal function tests, myocardial enzymes, serum electrolytes, lactate dehydrogenase (LDH), serum ferritin (SF), and M. pneumoniae-specific immunoglobulin M antibodies (MP-IgM).

### 2.3 Bronchoscopy and BALF collection

After entering the bronchoscopy suite, children received sedation with midazolam. As the bronchoscope was advanced, topical anesthesia was achieved by spraying 1% or 2% lidocaine onto the larynx, the area just above the vocal cords, and the carina. The lavage site was selected based on chest imaging findings, targeting the most severely affected pulmonary segment or lobe. After the bronchoscope was wedged into the target bronchus, sterile normal saline prewarmed to 37°C was instilled through the working channel in aliquots to a total volume of 1–2 mL/kg, and at least 40% of the instilled fluid was recovered.

A portion of the BALF was immediately sent for routine testing, including differential cell counts and detection of *M. pneumoniae* RNA. An additional 3 mL of BALF was processed within 2 h of collection. Samples were centrifuged at 4°C to remove cells and debris, aliquoted, and stored at −80°C until analysis.

### 2.4 Measurement of BALF cytokines, epithelial injury biomarkers, and mucins

Concentrations of cytokines (IL-2, IL-4, IL-6, IL-10, IL-17, interferon-γ [IFN-γ], IL-12p70, IL-1β, and tumor necrosis factor-α [TNF-α]), epithelial injury markers [Krebs von den Lungen-6 (KL-6), surfactant protein A (SP-A), surfactant protein D (SP-D), and short palate, lung, and nasal epithelium clone 1 (SPLUNC1)], and mucins (MUC1, MUC4, MUC5AC, MUC5B, and MUC16) in BALF were quantified using commercially available sandwich enzyme-linked immunosorbent assay (ELISA) kits (Bioswamp, Wuhan, China), according to the manufacturer’s instructions. Concentrations were calculated using standard curves.

### 2.5 Detection of macrolide-resistance gene mutations

BALF specimens were analyzed using a fluorescence polymerase chain reaction (PCR) kit (Mole Biotechnology, Jiangsu, China) targeting *M. pneumoniae* nucleic acids and 23S rRNA macrolide-resistance mutations at positions 2063 (A→G) and 2064 (A→G). Detection of a mutation at either site was interpreted as macrolide resistance.

### 2.6 Statistical analysis

Data were analyzed using SPSS software (version 22.0) and R software (version 4.4.0). Normally distributed continuous variables are presented as mean ± SD and were compared using independent-sample t-tests, whereas non-normally distributed variables are presented as median (P25, P75) and were compared using Mann–Whitney U tests. Categorical variables are presented as n (%) and were analyzed using Fisher’s exact test. Correlations were assessed using Spearman correlation analysis. Differentially expressed BALF cytokines and mucins were further analyzed using GO and KEGG enrichment analyses. Variables showing significant associations with mucus plugs in univariable analyses, together with clinically important variables, were entered into a least absolute shrinkage and selection operator (LASSO) logistic regression model to select key features. Seven selected features were subsequently included in a multivariable logistic regression model.

## 3 Results

### 3.1 Participant flow and cohort formation

**Figure 1:**
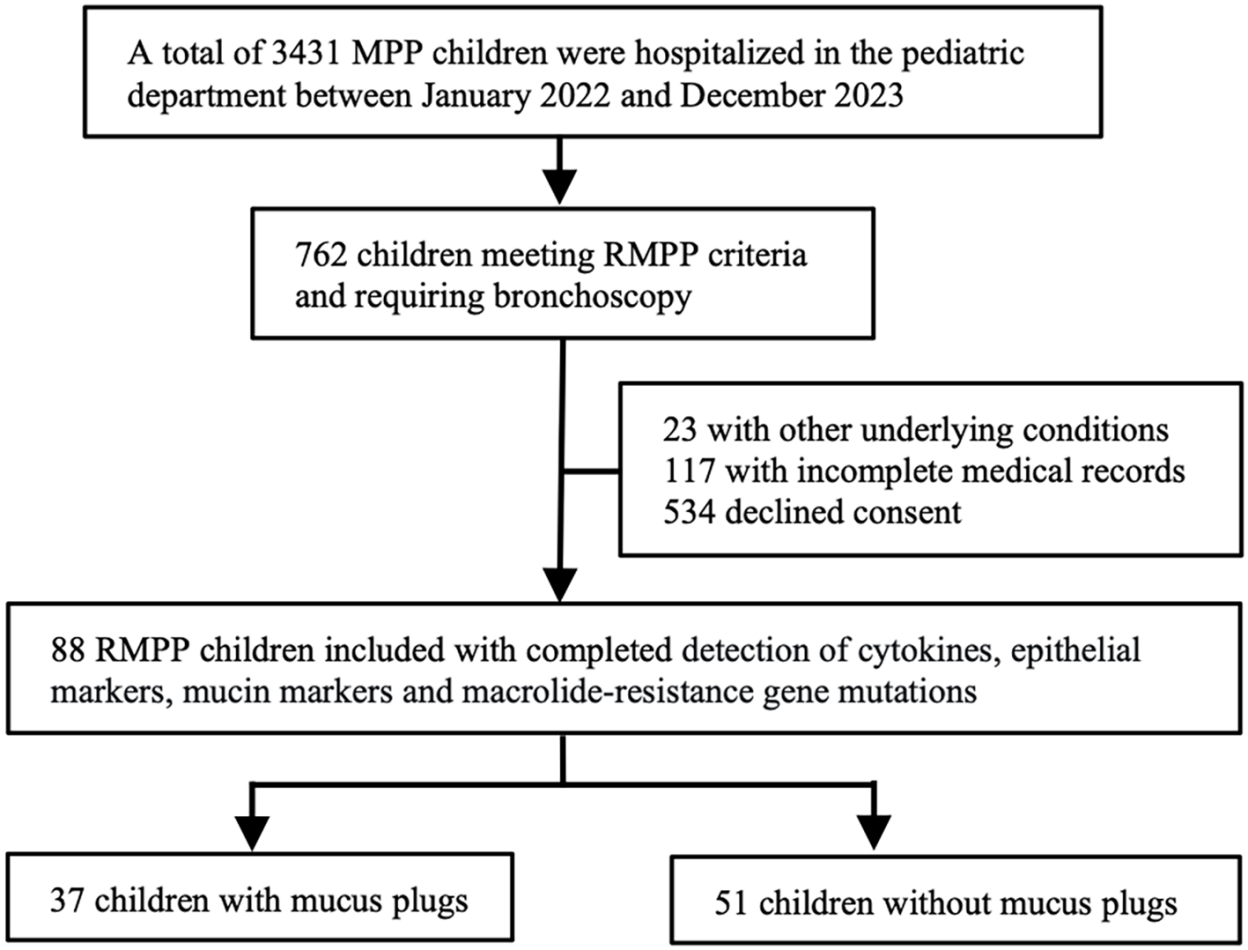
Flow chart of patient inclusion and exclusion.

Between January 2022 and December 2023, a total of 3,431 children were hospitalized with MPP. Among these patients, 762 met the criteria for RMPP and required bronchoscopy. After excluding 23 children with comorbidities, 117 with missing clinical data, and 534 whose guardians declined participation, 88 children were ultimately enrolled. Based on bronchoscopic findings, 37 children were classified into the mucus plug group and 51 into the non–mucus plug group. A detailed flow diagram of participant selection is shown in **Figure 1**.

### 3.2 Baseline clinical and serologic characteristics

**Figure 2:**
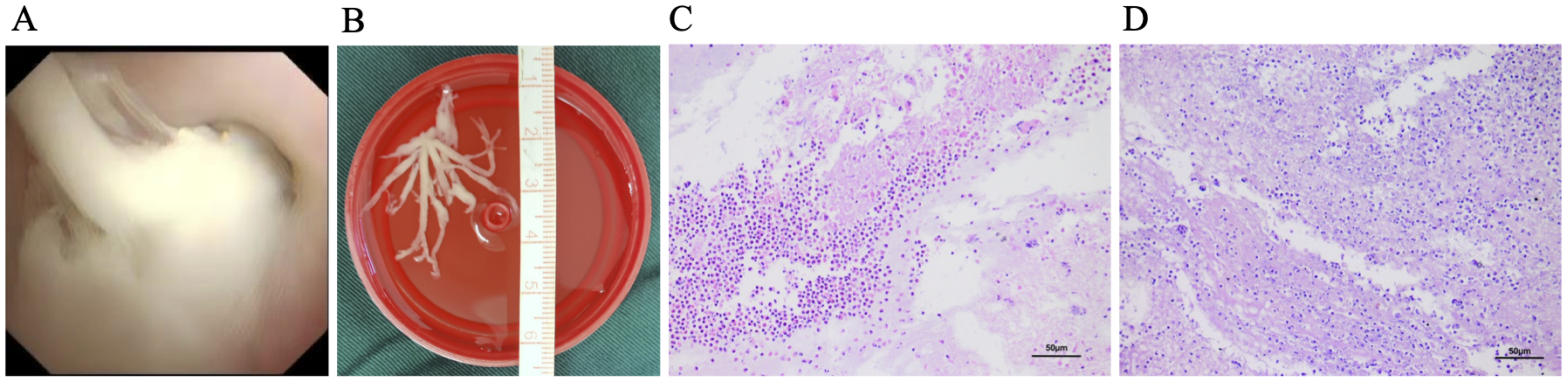
Mucus plugs in RMPP: gross and histopathologic features. **(A)** Bronchoscopic appearance of mucus plugs in RMPP. **(B)** Gross appearance of mucus plugs retrieved by bronchoscopy. **(C)** H&E staining of a mucus plug. **(D)** PAS staining highlighting mucin within the plug.

Bronchoscopic examination demonstrated tenacious secretions occluding segmental and subsegmental bronchi (**Figure 2A**). After removal, these plugs appeared ex vivo as strip- or cast-shaped specimens (**Figure 2B**). Histopathologic examination using hematoxylin–eosin (H&E) and periodic acid–Schiff (PAS) staining revealed mucinous material with abundant fibrin and a dense, neutrophil-predominant inflammatory infiltrate (**Figures 2C, D**).

The mucus plug group included a higher proportion of male patients, more frequently underwent repeat bronchoscopy and bronchial brushing, and more often required the use of biopsy forceps (**Table 1**). However, no significant differences were observed between groups with respect to age, history of eczema or allergy, peak temperature, duration of fever or cough, presence of wheezing, lung auscultation findings (crackles, rhonchi, wheezes, or decreased breath sounds), pulmonary consolidation, pleural effusion, or extrapulmonary involvement (**Supplementary Table S1**).

**Table 1:** Baseline characteristics, laboratory, and serologic parameters of RMPP patients with and without mucus plugs.

Variable	Mucus plug group (n = 37)	Non-mucus plug group (n = 51)	*p*
Clinical characteristics			
Sex, male, n (%)	28 (75.7%)	28 (54.9%)	0.046
Repeat bronchoscopy, n (%)	4 (10.8%)	0 (0%)	0.017
Bronchial brushing, n (%)	27 (73.0%)	4 (7.8%)	0.000
Biopsy forceps utilization, n (%)	18 (48.6%)	1 (2.0%)	0.000
Laboratory and serological parameters			
L (×10^9^/L)	1.3 (0.8, 2.0)	1.85 (1.2, 3.0)	0.010
M (×10^9^/L)	0.6 (0.3, 0.9)	0.75 (0.5, 1.1)	0.030
PLT (×10^9^/L)	284.2 ± 83.4	361.9 ± 117.4	0.001
CRP (mg/L)	39.7 (14.36, 78.1)	17.55 (8.1, 45.1)	0.024
PCT (ng/mL)	0.15 (0.12, 0.23)	0.11 (0.07, 0.19)	0.029
Total protein (g/L)	61.4 ± 5.1	65.8 ± 5.2	0.000
Albumin (g/L)	35.2 ± 3.8	37.1 ± 4.1	0.029
Na^+^ (mmol/L)	136.5 ± 2.9	138.4 ± 2.8	0.004
K^+^ (mmol/L)	4.1 ± 0.4	4.3 ± 0.4	0.031
CK (U/L)	69.5 (43.8, 106.3)	39 (24.3, 60.8)	0.001
LDH (U/L)	494 (327.5, 628)	350 (280.3, 460)	0.008
SF (ng/mL)	354.6 (211.1, 456)	145.6 (106.5, 316.9)	0.002

L, lymphocyte count; M, monocyte count; PLT, platelet count; CRP, C-reactive protein; PCT, procalcitonin; Na⁺, sodium; K⁺, potassium; CK, creatine kinase; LDH, lactate dehydrogenase; SF, serum ferritin.

Children in the mucus plug group exhibited lower lymphocyte counts (1.3 *vs*. 1.85 ×10^9^/L; *p* = 0.010), monocyte counts (0.6 *vs*. 0.75 ×10^9^/L; *p* = 0.030), platelet counts (284.2 ± 83.4 *vs*. 361.9 ± 117.4 ×10^9^/L; *p* = 0.001), total protein levels (61.4 ± 5.1 *vs*. 65.8 ± 5.2 g/L; *p* < 0.0001), albumin levels (35.2 ± 3.8 *vs*. 37.1 ± 4.1 g/L; *p* = 0.029), sodium levels (136.5 ± 2.9 *vs*. 138.4 ± 2.8 mmol/L; *p* = 0.004), and potassium levels (4.1 ± 0.4 *vs*. 4.3 ± 0.4 mmol/L; *p* = 0.031). In contrast, markers of systemic inflammation were significantly higher, including (CRP; 39.7 *vs*. 17.55 mg/L; *p* = 0.024), (PCT; 0.15 *vs*. 0.11 ng/mL; *p* = 0.029), creatine kinase (CK; 69.5 *vs*. 39.0 U/L; *p* = 0.001), (LDH; 494 *vs*. 350 U/L; *p* = 0.008), and (SF; 354.6 *vs*. 145.6 ng/mL; *p* = 0.002) (**Table 1**). No significant differences were observed in total white blood cell count, absolute neutrophil count, D-dimer, serum chloride (Cl^-^), or creatine kinase–MB (CK-MB) levels (*p* > 0.05; **Supplementary Table S1**).

### 3.3 Comparison of cytokines, epithelial markers, and mucins in BALF between groups

Cytokines in BALF are secreted predominantly by resident immune cells (e.g., alveolar macrophages, lymphocytes, and neutrophils), as well as airway epithelial cells, and therefore more directly reflect the pulmonary microenvironment than serum markers. Compared with the non–mucus plug group, children with mucus plugs exhibited significantly higher BALF levels of IL-2 (4.01 *vs*. 3.21 pg/mL; *p* = 0.020), IL-4 (7.62 *vs*. 3.65 pg/mL; *p* = 0.003), IL-6 (164.79 *vs*. 124.69 pg/mL; *p* = 0.011), IL-17 (13.16 *vs*. 6.64 pg/mL; *p* = 0.014), (IFN-γ; 42.75 *vs*. 18.49 pg/mL; *p* = 0.010), IL-12p70 (5.40 *vs*. 3.33 pg/mL; *p* = 0.012), and (TNF-α; 23.7 *vs*. 11.6 pg/mL; *p* = 0.031). Data are presented as medians (interquartile range). This pattern suggests an upregulated local inflammatory milieu involving Th1, Th2, and Th17 immune pathways in the mucus plug group (**Table 2**). No significant differences were observed in IL-10 or IL-1β levels (*p* > 0.05; **Table 2**).

**Table 2:** BALF cytokines, epithelial biomarkers, and mucins in RMPP patients with and without mucus plugs.

Variable	Mucus plug group (n = 37)	Non-mucus plug group (n = 51)	*p*
BALF cytokines			
IL-2 (pg/mL)	4.01 (3.0, 7.5)	3.21 (1.9, 5.3)	0.020
IL-4 (pg/mL)	7.62 (3.6, 12.9)	3.65 (1.6, 7.1)	0.003
IL-6 (pg/mL)	164.79 (89. 8, 507.4)	124.69 (47.8, 200.9)	0.011
IL-17 (pg/mL)	13.16 (6.8, 25.3)	6.64 (3.3, 14.2)	0.014
IFN-γ (pg/mL)	42.75 (15.6, 89.4)	18.49 (7. 9, 48.5)	0.010
IL-12p70 (pg/mL)	5.4 (3.2, 9.3)	3.33 (1.8, 6.7)	0.012
TNF-α (pg/mL)	23.7 (12.9, 58.3)	11.6 (5.2, 41.7)	0.031
IL-10 (pg/mL)	5.72 (2.13, 12.8)	3.69 (1.9, 12.1)	0.603
IL-1β (pg/mL)	1096.8 (297, 2548.8)	624.3 (175.6, 1952.4)	0.159
BALF epithelial biomarkers			
SP-A (ng/mL)	33.4 (17.2, 41.3)	19.3 (15.3, 36.2)	0.023
KL-6 (U/mL)	469.6 ± 85.1	432.9 ± 82.9	0.046
SP-D (ng/mL)	11.4 ± 2.8	10.5 ± 2.3	0.121
SPLUNC1 (ng/mL)	1.5 ± 0.4	1.4 ± 0.4	0.232
BALF mucins			
MUC5B (ng/mL)	4.6 (4.1, 5)	4.2 (3.9, 4.7)	0.035
MUC1 (ng/mL)	16.9 (10.7, 20.7)	12.3 (10.7, 22.3)	0.777
MUC4 (ng/mL)	11.9 (4.8, 13.3)	5.6 (4.3, 14)	0.432
MUC5AC (pg/mL)	1178.2 ± 211.6	1116.4 ± 180.4	0.144
MUC16 (pg/mL)	240.9 (220.2, 270.4)	248.8 (224.6, 273.2)	0.475

Epithelial injury markers, including Krebs von den Lungen-6 (KL-6), SP-A, SP-D, SPLUNC1, are primarily secreted by alveolar type II epithelial cells and airway epithelial cells and serve as indicators of epithelial integrity, barrier function, and surfactant homeostasis (Jaiswal et al., 2022; Heltborg et al., 2024). In this study, children in the mucus plug group exhibited higher levels of epithelial injury markers, including SP-A (33.4 *vs*. 19.3 ng/mL; *p* = 0.023) and KL-6 (469.6 ± 85.1 *vs*. 432.9 ± 82.9 U/mL; *p* = 0.046) (**Table 2**). No significant differences were observed in SP-D or SPLUNC1 levels (**Table 2**).

Mucin markers, including MUC1, MUC4, MUC5AC, MUC5B, and MUC16, are major components of airway mucus that contribute to epithelial protection and mucociliary clearance (Ma et al., 2018). Dysregulated mucin expression alters mucus rheology, impairs clearance, and promotes airway mucus plug formation (Liegeois et al., 2025). In this cohort, BALF levels of MUC5B were significantly higher in the mucus plug group (4.6 *vs*. 4.2 ng/mL; *p* = 0.035). No significant differences were observed in MUC1, MUC4, MUC5AC, or MUC16 levels (**Table 2**).

### 3.4 Comparison of resistance gene mutations and BALF cellular composition

No significant difference was observed in the prevalence of macrolide-resistance gene mutations between the mucus plug and non–mucus plug groups (100% *vs*. 98%; *p* = 0.411). Similarly, BALF cellular composition—including segmented neutrophils, lymphocytes, band neutrophils, macrophages, and epithelial cells—did not differ significantly between groups (*p* > 0.05; **Supplementary Table S2**).

### 3.5 Correlations linking systemic and airway markers

**Figure 3:**
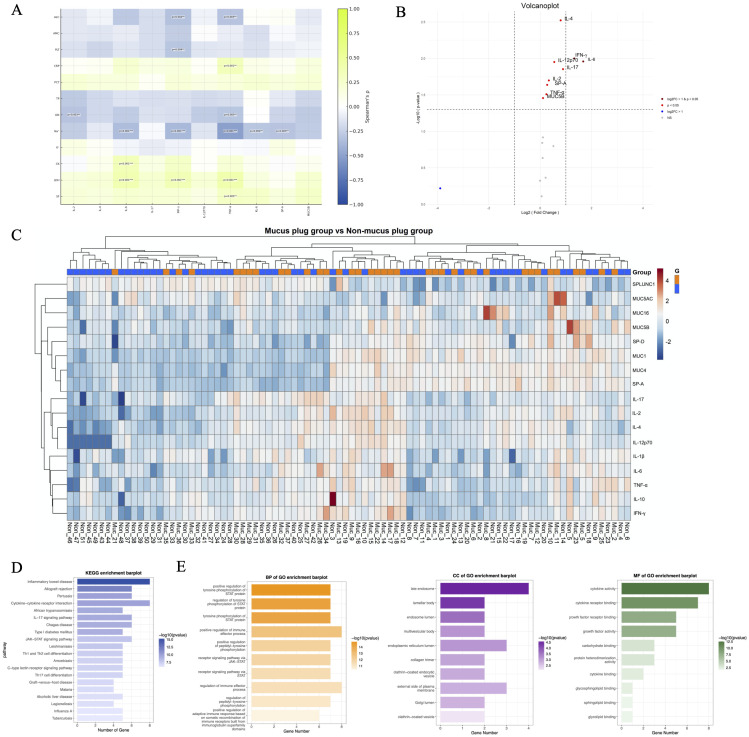
Integrated analysis of bronchoalveolar lavage fluid (BALF) markers: pairwise correlations, differential expression, protein heatmap, and GO/KEGG pathway enrichment. **(A)** Pairwise associations between systemic biomarkers and airway markers in BALF. Heatmap of Spearman correlation coefficients (ρ) across all marker pairs; colors indicate direction and magnitude (blue, negative; green, positive). Asterisks denote false discovery rate (FDR)–adjusted significance (*q* < 0.05); *p*-values shown in the heatmap are raw (unadjusted) and provided for reference. **(B)** Volcano plot of differentially expressed molecules between groups. The x-axis represents log_2_ fold change (positive values indicate higher abundance in the mucus plug group), and the y-axis represents −log_10_(*p*). **(C)** Heatmap of BALF proteins in mucus plug and non–mucus plug groups. Columns represent individual patients, and rows represent mucins, epithelial proteins, and cytokines. Values were log_10_-transformed and row-wise z-scored; both rows and columns were hierarchically clustered. **(D)** Kyoto Encyclopedia of Genes and Genomes (KEGG) pathway enrichment analysis. **(E)** Gene Ontology (GO) enrichment analysis of biological process (BP), molecular function (MF), and cellular component (CC) terms.

Spearman correlation analyses were performed between serologic indicators and BALF markers that showed significant intergroup differences. The resulting heatmap revealed a coordinated serum–BALF pattern. Serum CRP, LDH, CK, and SF were positively correlated with BALF pro-inflammatory cytokines (IL-6, IFN-γ, and TNF-α). Specifically, CRP correlated positively with TNF-α (ρ = 0.34, *p* = 0.001); LDH correlated positively with IL-6 (ρ = 0.39, *p* < 0.001), IFN-γ (ρ = 0.36, *p* < 0.001), and TNF-α (ρ = 0.41, *p* < 0.001); CK correlated positively with IL-6 (ρ = 0.36, *p* < 0.001); and SF correlated positively with TNF-α (ρ = 0.29, *p* = 0.005). In parallel, epithelial injury markers and mucins aligned with this inflammatory axis: KL-6 and SP-A increased in concert with the inflammatory marker cluster (CRP, PCT, LDH, and SF), while MUC5B clustered within the same positive module (**Figure 3A**).

Conversely, several serologic markers exhibited inverse associations with airway inflammation and epithelial injury (**Figure 3A**). Serum albumin was negatively correlated with IL-2 (ρ = −0.31, *p* = 0.003) and TNF-α (ρ = −0.30, *p* = 0.005). Serum sodium showed consistent inverse correlations with IL-6 (ρ = −0.35, *p* < 0.001), IFN-γ (ρ = −0.37, *p* < 0.001), TNF-α (ρ = −0.50, *p* < 0.001), KL-6 (ρ = −0.30, *p* = 0.004), and SP-A (ρ = −0.31, *p* = 0.003). Platelet counts were also inversely correlated with IL-6 (ρ = −0.13, *p* = 0.004). Lymphocyte counts demonstrated inverse associations with IFN-γ (ρ = −0.32, *p* = 0.002) and TNF-α (ρ = −0.31, *p* = 0.003). Taken together, these correlations indicate that lower lymphocyte counts, albumin levels, electrolyte concentrations, and platelet counts are associated with heightened local airway inflammatory activity.

### 3.6 Integrated analysis of BALF markers

The volcano plot demonstrated a right-shifted distribution, indicating a greater number of upregulated than downregulated analytes. IL-6 and IFN-γ met the joint significance threshold (|log_2_ fold change| > 1 and *p* < 0.05). IL-2, IL-4, IL-12p70, IL-17, SP-A, TNF-α, and MUC5B reached statistical significance (*p* < 0.05) but exhibited smaller effect sizes (|log_2_ fold change| ≤ 1) (**Figure 3B**). No analytes met the predefined criteria for downregulation. Heatmap visualization revealed that a subset of mucus plug cases exhibited concordant elevations in mucin and epithelial markers—particularly MUC5B, MUC16, SP-A, and SP-D—accompanied by higher levels of multiple pro-inflammatory cytokines, most notably IL-6, IL-1β, and IL-17. In contrast, non–mucus plug cases showed comparatively lower expression levels of these proteins. Hierarchical clustering grouped mucin and epithelial markers with inflammatory cytokines into adjacent modules, indicating covariation between mucus secretion, epithelial responses, and airway inflammatory activation (**Figure 3C**).

Differentially expressed BALF cytokines, epithelial injury biomarkers, and mucin markers were subjected to GO and KEGG enrichment analyses. KEGG pathway analysis demonstrated enrichment in cytokine–cytokine receptor interaction, IL-17 signaling, T-helper cell differentiation (Th1/Th2/Th17), and multiple infection- and inflammation-related pathways (**Figure 3D**), all of which are closely linked to immune activation and chronic inflammation. GO analysis highlighted Janus kinase–signal transducer and activator of transcription (JAK–STAT)-centered immune signaling. Top BP terms included positive regulation of tyrosine phosphorylation of STAT proteins, receptor signaling via the JAK–STAT pathway, and positive regulation of immune effector processes. MF terms were dominated by cytokine-related activities, including growth factor activity and receptor binding, indicating amplified inflammatory signaling. CC terms mapped primarily to secretory and vesicular trafficking structures, as well as lamellar bodies—surfactant-producing organelles of type II pneumocytes—consistent with enhanced epithelial secretion and mucin transport (**Figure 3E**).

### 3.7 Logistic regression model for mucus plug formation.

**Figure 4:**
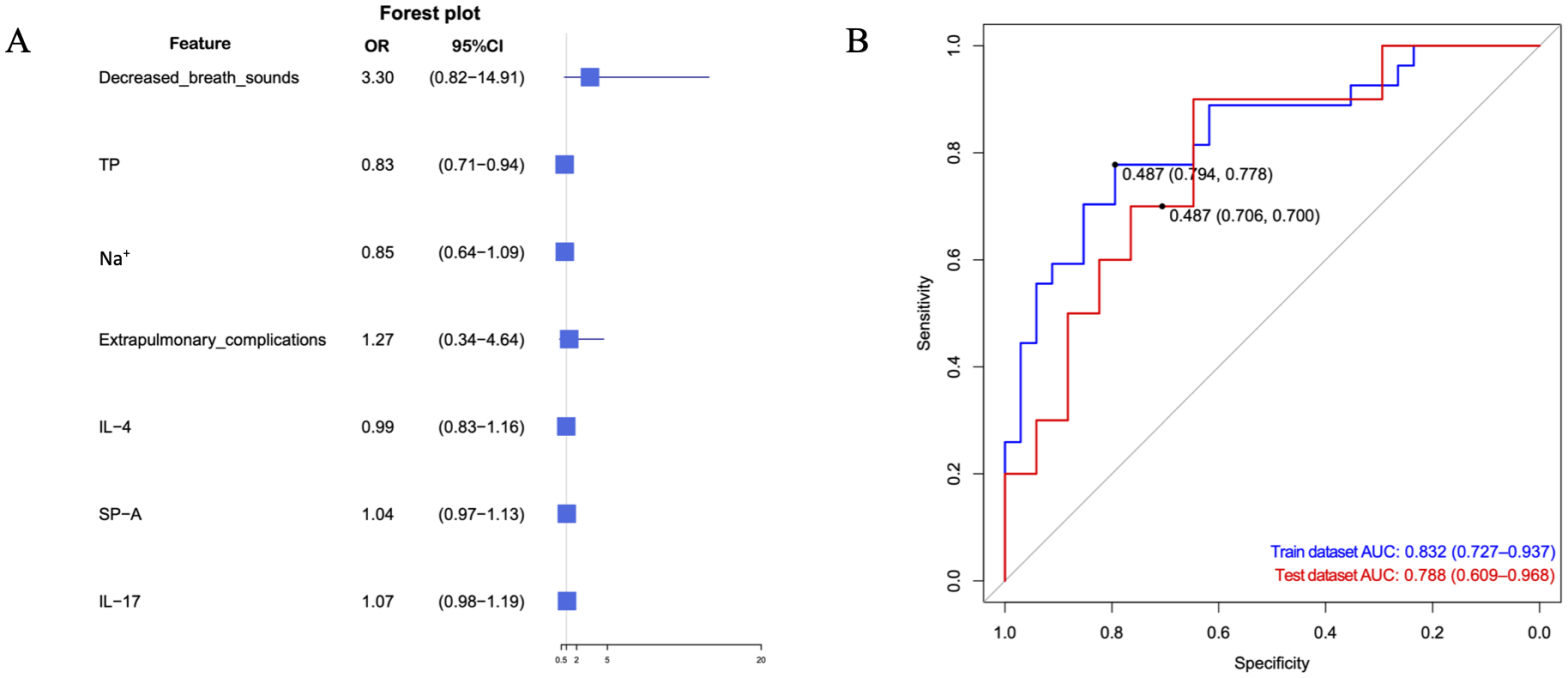
Multivariable logistic regression analysis of factors associated with mucus plug formation. **(A)** Forest plot showing adjusted odds ratios (ORs) with 95% confidence intervals (CIs) for least absolute shrinkage and selection operator (LASSO)–selected predictors, including decreased breath sounds, total protein (TP), sodium (Na^+^), extrapulmonary complications, IL-4, surfactant protein A (SP-A), and IL-17. **(B)** Receiver operating characteristic (ROC) curves for the final logistic regression model predicting mucus plug formation. Axes depict sensitivity versus 1 − specificity; the diagonal line indicates no discrimination.

LASSO regression retained seven predictors for inclusion in the multivariable logistic regression model. Among these variables, only serum total protein demonstrated an independent association with mucus plug formation (adjusted odds ratio [aOR], 0.83; 95% confidence interval [CI], 0.71–0.94). The remaining predictors showed directionally consistent associations but were imprecise, with wide confidence intervals (**Figure 4A**). Model discrimination was moderate, with an area under the receiver operating characteristic curve (AUC) of 0.83 in the training set and 0.79 in the test set (**Figure 4B**), and model calibration was acceptable. Cross-validation curves and the nomogram are provided in **Supplementary Figure S1**. This model is presented as mechanistic support rather than as a clinical prediction tool and requires external validation.

## 4 Discussion

In recent years, the incidence of RMPP has increased steadily. Bronchial mucus plugs, a major complication and risk factor in RMPP, may progress to plastic bronchitis and become life-threatening (Zhang et al., 2023). In this study, the mucus plug group had a higher proportion of male patients and more frequently required bronchial brushing, use of biopsy forceps, and repeat bronchoscopy, reflecting the tenacious and obstructive nature of these plugs. In contrast, other baseline clinical characteristics showed no significant differences between groups. These findings suggest that clinical features alone are insufficient to predict mucus plug formation, highlighting the need for more precise laboratory-based indicators.

Our results demonstrated that children with mucus plugs exhibited higher levels of systemic inflammatory markers and multiple local BALF cytokines. Serum markers, including CRP, PCT, LDH, and SF, were significantly elevated in the mucus plug group, reflecting a pronounced systemic inflammatory response. CRP and PCT are sensitive indicators of infection severity in RMPP (Xu et al., 2017; Gu et al., 2025), and elevated CRP has previously been reported as a predictor of mucus plug formation in pediatric MPP (Xu et al., 2017; Li et al., 2025a). LDH levels increase following pulmonary infection and tissue necrosis as intracellular enzymes are released, and both LDH and SF have been shown to be significantly elevated in patients with MPP complicated by necrotizing pneumonia (Li et al., 2023; Wang et al., 2023). Notably, prior studies have identified LDH and SF as independent risk factors for mucus plug formation in RMPP (Li et al., 2025a). BALF cytokine profiling further revealed marked localized immune activation within the airways. In the present study, multiple cytokines were significantly elevated in the mucus plug group, including pro-inflammatory mediators (IL-6, TNF-α, and IFN-γ), a key regulator of mucus secretion (IL-4), and cytokines associated with tissue injury and fibrotic processes (IL-17 and IL-12p70). These findings indicate an exaggerated local immune response involving Th1, Th2, and Th17 pathways. The concurrent activation of these immune axes suggests an amplified inflammatory cascade that may promote epithelial injury, excessive mucin secretion, and ultimately mucus plug formation.

Concurrently, epithelial injury markers, including KL-6 and surfactant protein A (SP-A), were markedly elevated in children with mucus plugs. KL-6, synthesized and secreted by alveolar type II epithelial cells (AEC-II) (Gao et al., 2024), increases in BALF following epithelial injury and disruption of the alveolar–capillary barrier. Such injury may facilitate mucus hypersecretion and impair mucociliary clearance, thereby promoting mucus plug formation (Piazza et al., 2022; Jehn et al., 2023; Gao et al., 2024). SP-A, produced by AEC-II and Clara cells and secreted into the alveolar lumen, plays a critical role in surfactant structure, function, and metabolism (Li et al., 2025c). In addition, SP-A contributes to innate immune defense by inhibiting pathogen growth and binding microbial ligands to enhance clearance by alveolar macrophages (Watson et al., 2020; Di et al., 2024). Collectively, the elevated levels of KL-6 and SP-A observed in this study highlight substantial epithelial injury and compromised airway defense in RMPP patients with mucus plugs.

Following the evidence of epithelial injury, we examined the contribution of mucins to mucus plug formation. Pulmonary mucus is primarily composed of the gel-forming mucins MUC5B and MUC5AC, which differ in glycosylation patterns and macromolecular assembly (Ma et al., 2018). In healthy airways, mucus is predominantly composed of MUC5B, with relatively lower levels of MUC5AC. Functionally, MUC5B is essential for maintaining appropriate mucus viscoelasticity and effective mucociliary clearance, thereby supporting host defense through the trapping and removal of pathogens (Ma et al., 2018; Huang et al., 2022). Consistently, loss-of-function models and human cases with deficient MUC5B exhibit recurrent bacterial infections, impaired mucociliary clearance, and granulocytic accumulation, underscoring its role in airway defense and immune homeostasis (Costain et al., 2022; Liegeois and Fahy, 2022).

By contrast, disease states shift this balance in condition-specific ways. In asthma, the mucin profile tends toward MUC5AC dominance (Liegeois et al., 2025). In chronic obstructive pulmonary disease (COPD), a multicenter observational study reported higher sputum levels of both mucins than in healthy controls, with a predominant increase in MUC5AC. Moreover, mean MUC5AC—but not MUC5B—levels were higher in participants experiencing exacerbations. Consistently, sputum MUC5AC correlates more strongly with small-airway abnormalities, airflow obstruction, and exacerbation risk and is more responsive to smoking-related stimuli, whereas MUC5B shows weaker and less consistent associations with clinical phenotypes (Radicioni et al., 2021). Taken together, findings from the asthma and COPD literature converge on a model in which MUC5AC is more closely linked to disease pathology, whereas MUC5B plays a comparatively lesser role.

In pediatric MPP, mucin characterization remains limited. One study reported that both MUC5AC and MUC5B levels in BALF were higher in children with MPP than in those with foreign body aspiration (Ma et al., 2022). Extending these observations, our cohort demonstrated higher airway MUC5B—but not MUC5AC—levels in children with mucus plugs. These findings are not contradictory. Infection-related stimuli may elevate both mucins overall; however, within the RMPP mucus plug phenotype, the relative signal appears shifted toward MUC5B. This pattern suggests that MUC5B upregulation is a characteristic feature of this subtype and adds to the current understanding of airway mucin composition in RMPP. Notably, this profile differs from the mucin signatures typically observed in asthma and COPD, raising the possibility that mucus plug formation in RMPP follows a distinct pathophysiological mechanism.

Mechanistically, overlapping inflammatory cues can induce both mucins. Th2 cytokines (e.g., IL-13) and non-Th2 cytokines (IL-1β, IL-17A, and TNF-α) upregulate MUC5AC expression (Zhang et al., 2019; Ye et al., 2025), whereas mixed T-helper cytokines, including IL-4, IL-13, IL-17, and TNF-α, can drive MUC5B overexpression (Zhang et al., 2019; Mammen et al., 2021; Kumar et al., 2023; Seo et al., 2023). Despite these shared upstream signals, their roles diverge in disease contexts. In murine asthma models, *Muc5ac* deletion reduces allergen-induced mucus plugging and airway hyperresponsiveness, indicating that MUC5AC is required for these pathological features (Evans et al., 2015), whereas *Muc5b* deficiency does not confer comparable protection (Raclawska et al., 2016). In our cohort, within a mixed Th1/Th2/Th17 inflammatory milieu, selective MUC5B upregulation likely reflects an infection-driven epithelial response. We speculate that two processes may coexist. First, compensatory MUC5B secretion may enhance pathogen trapping and mucociliary clearance. Second, under conditions of high inflammatory burden and epithelial barrier disruption, excessive MUC5B expression may stabilize mucus networks and promote mucus cast formation, thereby sustaining airway obstruction. This interpretation is partially supported by *in vitro* evidence demonstrating that *M. pneumoniae* factors co-induce MUC5B expression and pro-inflammatory cytokines, including IL-6 and TNF-α, in airway epithelial cells (Ma et al., 2022). Taken together, these observations raise the possibility that MUC5B functions as a context-dependent mediator—supporting host defense under infectious pressure but, when excessive, contributing to mucus plug persistence in RMPP.

Macrolide resistance has emerged as a major driver of treatment failure in pediatric MPP, contributing to prolonged fever, delayed radiographic resolution, and progression to refractory phenotypes (Wang et al., 2024). A recent study identified macrolide-resistant *M. pneumoniae* (MRMP) as an independent risk factor for mucus plug formation (Chunlian et al., 2025). Resistance rates vary geographically, with global prevalence estimated at approximately 61% and particularly high rates reported in East Asia (68% in China, 61% in Japan, and 63% in South Korea) (Yang et al., 2025). MRMP has also been associated with higher proportions of severe disease (95%), refractory cases (95%), and mucus plug formation (95%) (Yang et al., 2025). In the present cohort, macrolide resistance did not differ significantly between groups, likely reflecting selection bias. By design, patients were enrolled after macrolide treatment failure, resulting in near-universal resistance (mutation rates of 100% in the mucus plug group and 98% in the non–mucus plug group). Consequently, macrolide resistance could not serve as a discriminating variable in this analysis, although its contributory role in disease pathogenesis cannot be excluded. We therefore interpret macrolide resistance as a probable upstream driver of refractoriness, whereas exaggerated inflammation and epithelial injury may represent the key determinants of mucus plug formation among children with RMPP.

Correlation analyses demonstrated coordinated links between systemic indicators and the local airway environment. LDH, CRP, and CK correlated positively with BALF pro-inflammatory cytokines (IL-6, interferon-γ [IFN-γ], and tumor necrosis factor-α [TNF-α]), whereas the serologic markers albumin and sodium showed consistent inverse correlations with multiple BALF cytokines (IL-2, IL-6, IFN-γ, and TNF-α) and with epithelial injury markers (KL-6 and SP-A). KEGG enrichment analysis indicated that differentially expressed cytokines and mucins were involved in multiple inflammation-related pathways, and GO analysis coherently highlighted regulation of immune effector processes. Collectively, these findings suggest that mucus plug formation in RMPP is associated with exaggerated systemic and local immune responses.

In the multivariable model, only serum total protein remained independently associated with mucus plug formation, despite multiple predictors reaching significance in univariable analyses. This finding should be interpreted with caution. Serum total protein, which is largely influenced by albumin, is a negative acute-phase reactant and may primarily reflect overall inflammatory burden, capillary leak, and nutritional status rather than acting as a direct mechanistic driver of mucus plug formation. In children with RMPP, intense and prolonged systemic inflammation may reduce hepatic protein synthesis and increase transvascular protein leakage into the lung, thereby lowering circulating protein levels while simultaneously contributing to protein-rich airway exudates. Accordingly, low serum total protein likely represents an integrated marker of disease severity and epithelial–endothelial barrier dysfunction rather than a specific causal factor. The regression model is therefore presented as hypothesis-supporting, in conjunction with the biological findings, rather than as a deployable bedside prediction tool.

This study has several limitations. First, it was a single-center study with a relatively small sample size, which limits generalizability and precludes causal inference. Second, as an observational study, it demonstrates associations rather than causation, and further experimental validation is required. Third, although we applied a bronchoscopic definition and a minimum extent criterion for mucus plugs, we did not employ an objective scoring system or formally assess inter-rater reliability; therefore, some degree of phenotype misclassification cannot be excluded. Finally, the exclusion of patients with incomplete clinical or laboratory data may have introduced selection bias, further limiting the generalizability of the identified risk factors and immune profiles. These findings should be validated in larger, multicenter cohorts.

Taken together, our results suggest that, beyond antimicrobial therapy, targeting excessive inflammation and abnormal mucus secretion may represent important adjunctive strategies for managing mucus plug formation in RMPP. Anti-inflammatory interventions, such as corticosteroids or other immunomodulatory agents, may help mitigate cytokine-associated epithelial injury. In addition, therapies aimed at regulating mucus properties—including mucolytic agents and strategies to limit excessive MUC5B production—may reduce the risk of obstructive plug formation. Future studies should focus on clarifying causal pathways through mechanistic investigations. Longitudinal sampling, external validation in multicenter cohorts, and omics-based profiling may further delineate the immune–epithelial–mucin axis and facilitate the identification of actionable therapeutic targets.

## 5 Conclusions

In this single-center cohort of pediatric RMPP, the mucus plug phenotype was accompanied by broader immune-inflammatory activity. Children with mucus plugs exhibited higher BALF cytokine levels spanning Th1, Th2, and Th17 pathways, elevated epithelial injury markers (KL-6 and SP-A), and relatively increased MUC5B. Cross-compartment analyses suggested coordinated relationships between systemic inflammation and the airway milieu, while multivariable modeling identified lower serum total protein as independently associated with mucus plug formation; other candidate variables showed directionally consistent associations but with imprecise estimates. Collectively, these findings delineate a pathophysiological context in RMPP in which epithelial injury and mucin enrichment coexist with heightened systemic inflammation. Targeted strategies aimed at modulating inflammatory responses and abnormal mucus secretion may offer new opportunities to improve clinical outcomes and prevent long-term pulmonary sequelae.

## Data Availability

The original contributions presented in the study are included in the article/**Supplementary Material**. Further inquiries can be directed to the corresponding authors.

## Supplementary material

Supplementary Figure 1 Nomogram and calibration of the predictive model for mucus plug formation in RMPP. **(A)** Calibration plot comparing predicted and observed probabilities of mucus plug formation. **(B)** Nomogram for estimating an individual child’s probability of having mucus plugs. For each predictor—decreased breath sounds, total protein (TP), sodium (Na^+^), extrapulmonary complications, IL-4, surfactant protein A (SP-A), and IL-17—the patient’s value is located to obtain points on the top scale. Points are summed to yield a value on the “Total Points” axis, which is then projected downward to estimate the corresponding disease probability.
