# Supplementary material for: Immune activation and mucin dysregulation in pediatric refractory Mycoplasma pneumoniae pneumonia with mucus plugs
Source: Front Cell Infect Microbiol. 2026 Jan 5;15:1706340. doi: 10.3389/fcimb.2025.1706340 (PMC12812597; doi:10.3389/fcimb.2025.1706340)
Supplement: Supplementary file 1 [file Table1.docx]

Supplementary Table S1. Additional baseline characteristics and serologic parameters of RMPP patients with and without mucus plugs.

| Variable | Mucus plug group  (n = 37) | Non-mucus plug group  (n = 51) | *p* |
| --- | --- | --- | --- |
| **Clinical characteristics** | | | |
| Age (years), n (%) | 7.8 ± 2.5 | 7.1 ±2 .2 | 0.190 |
| History of allergy, n (%) | 17 (45.9%) | 16 (31.4%) | 0.163 |
| Peak temperature (°C) | 40 (39, 40) | 40 (39, 40) | 0.429 |
| Duration of fever (days) | 10.9 ± 3.2 | 10.5 ± 4.3 | 0.621 |
| Duration of cough (days) | 19 (16.5, 23) | 19 (17, 22) | 0.579 |
| wheezing episodes, n (%) | 6 (16.2%) | 5 (10%) | 0.369 |
| Presence of crackles, n (%) | 26 (70.3%) | 32 (62.7%) | 0.462 |
| Presence of rhonchi, n (%) | 9 (24.3%) | 7 (13.7%) | 0.203 |
| Wheezing, n (%) | 8 (21.6%) | 5 (9.8%) | 0.123 |
| Decreased breath sounds, n (%) | 12 (32.4%) | 11 (21.6%) | 0.252 |
| Pulmonary consolidation, n (%) | 34 (91.9%) | 41 (80.4%) | 0.133 |
| Pleural effusion, n (%) | 20 (54.1%) | 18 (35.3%) | 0.079 |
| Extrapulmonary complications, n (%) | 19 (51.4%) | 18 (35.3%) | 0.132 |
| **Serological parameters** | | | |
| WBC (×10⁹/L) | 8.7 ± 5 | 10.2 ± 4.2 | 0.132 |
| N (×10⁹/L) | 6.1 ± 3.3 | 7 ± 3.6 | 0.222 |
| DD (ug/L) | 675 (349.5, 2241) | 395 (198, 1218) | 0.114 |
| Cl^-^ (mmol/L) | 103. 6± 3 | 103.6 ± 2.9 | 0.957 |
| CKMB (U/L) | 19 (14, 22.8) | 17 (14, 21.3) | 0.443 |

WBC, white blood cell count; N, neutrophil count; DD, D-dimer; Cl⁻, serum chloride; CKMB, creatine kinase MB isoenzyme.

Supplementary Table S2. Resistance gene mutations and BALF cellular composition between groups

| Variable | Mucus plug group  (n = 37) | Non-mucus plug group  (n = 51) | *p* |
| --- | --- | --- | --- |
| **Drug resistance** |  |  |  |
| Macrolide-resistance gene mutations | 34 (100%) | 50 (98%) | 0.411 |
| **BALF cellular composition** | | | |
| Segmented neutrophils (%) | 48.2 ± 21.4 | 45.8 ± 25 | 0.644 |
| Lymphocytes (%) | 8.5 (5, 17) | 9 (5, 18) | 0.705 |
| Band neutrophils (%) | 7 (4, 12.3) | 6.5 (3, 12.8) | 0.768 |
| Macrophages (%) | 28.6 ± 18.6 | 28.1 ± 22.1 | 0.913 |
| Epithelial cells (%) | 2 (1, 5.3) | 3 (2, 7) | 0.240 |
