# Supplementary figures and images for: Immune activation and mucin dysregulation in pediatric refractory Mycoplasma pneumoniae pneumonia with mucus plugs
Source: Front Cell Infect Microbiol. 2026 Jan 5;15:1706340. doi: 10.3389/fcimb.2025.1706340 (PMC12812597; doi:10.3389/fcimb.2025.1706340)

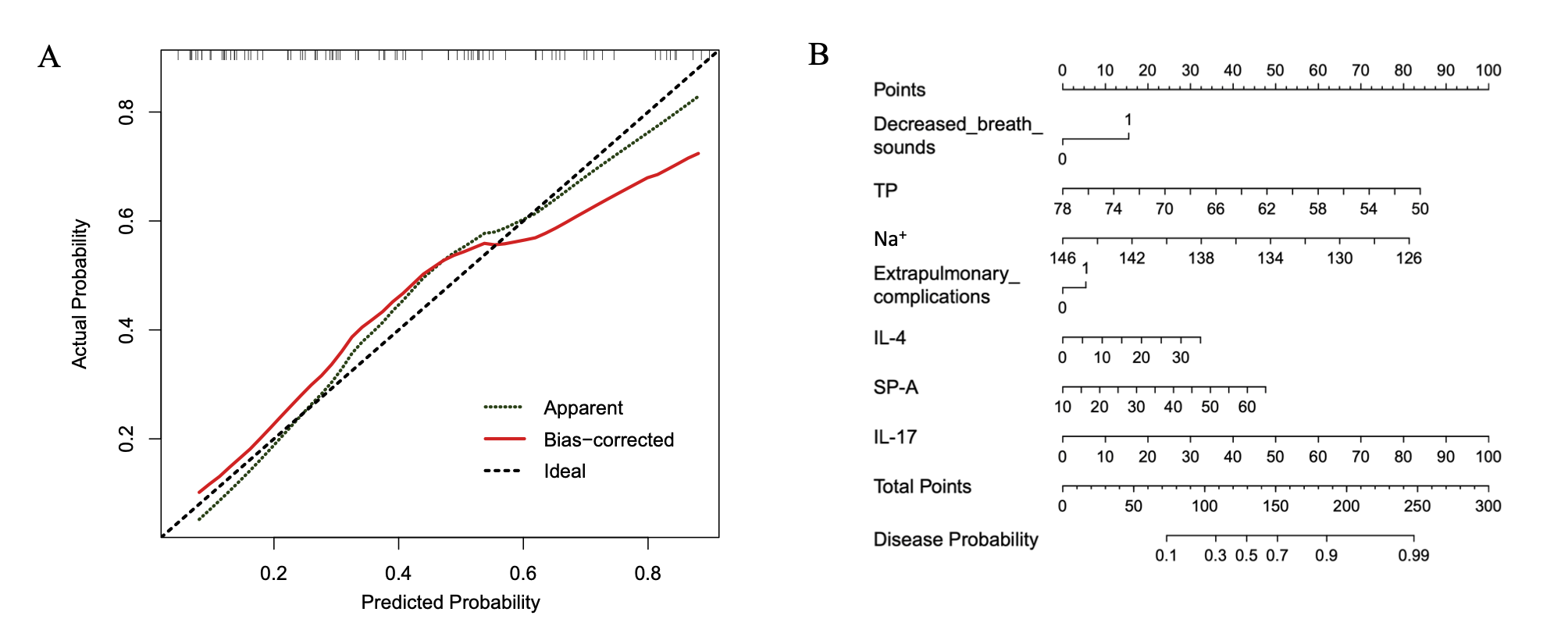

Supplement: Supplementary Figure 1 — Nomogram and calibration of the predictive model for mucus plug formation in RMPP. (A) Calibration plot comparing predicted and observed probabilities of mucus plug formation. (B) Nomogram for estimating an individual child’s probability of having mucus plugs. For each predictor—decreased breath sounds, total protein (TP), sodium (Na+), extrapulmonary complications, IL-4, surfactant protein A (SP-A), and IL-17—the patient’s value is located to obtain points on the top scale. Points are summed to yield a value on the “Total Points” axis, which is then projected downward to estimate the corresponding disease probability. [file Image1.tiff]
